# The complete mitochondrial genome of *Picromerus lewisi* Scott 1874 (Hemiptera: Pentatomidae: Asopinae)

**DOI:** 10.1080/23802359.2023.2179352

**Published:** 2023-02-20

**Authors:** Xiao-han Chen, Meng Leng, Di-hui Yao, Hai-yan Zhao, Mao-fa Yang

**Affiliations:** a College of Tobacco Science, Guizhou University, Guiyang, China; b Guizhou Provincial Key Laboratory of Tobacco Quality Research, Guiyang, China; c Institute of Entomology, Guizhou University, Guiyang, China

**Keywords:** *Picromerus lewisi* Scott, natural enemy, Asopinae, mitochondrial genome, phylogenetic analysis

## Abstract

*Picromerus lewisi* Scott (Hemiptera: Pentatomidae) is a widely used natural enemy, through this study, we proved that its complete mitochondrial genome of it had similar characteristics to those of other Hemiptera. The mitogenome of *P. lewisi* is a circular molecule of 18,123 bp with 74.0% A + T content, containing 13 protein-coding genes (PCGs), 22 tRNAs, 2 rRNAs, and one control region. Phylogenetic tree based on 13 PCGs from 17 Panheteroptera species (two species of the Cimicomorpha are used as outgroup, 15 species belong to the Pentatomomorpha) suggested that *P. lewisi* has a closer relationship with E. thomsoni within Pentatomidae family.

*Picromerus lewisi* Scott 1874 is an important generalist pentatomid predator of multiple species of pests, especially for lepidopteran pests (Tang et al. 2018; Wang et al. 2019, 2020; Fu et al. 2021; Shen et al. 2022), and has been used in biological control programs with its large predatory capacity, strong activity and adaptability in daily living, and suitable for artificial rearing (Yang et al. 2018). In this study, we sequenced the complete mitochondrial genome of *Picromerus lewisi* Scott. Surviving adult individuals were fed in the artificial climate chamber of Kaiyang country, Guiyang, Guizhou province of China (106°57′14′′E, 27°16′34′′N) (Figure 1). Presently, the sample was deposited in the Institute of Entomology, Guizhou University, Guiyang, China (voucher Gzh-2021-026, Hai-yan Zhao, haitianyiyan7611@163.com). We used the DNeasy Blood and Tissue Kit (cat.nos.69504 and 69506, QIAGEN, Hilden, Germany) to extract the total DNA from the head of the adult. An Illumina ReSeq library was produced, with an average insert size of 300 bp (a paired-end length of 150 bp). The library was then sequenced using the Illumina Novaseq6000 platform (Berry Genomics, Beijing, China). We assembled and annotated the complete mitogenome sequence using NOVOPlasty v2.7.2 (Dierckxsens et al. 2017), with the default setting for the K-mer value, and MitoZ v2.4-alpha (Meng et al. 2019) was used to annotate the genome.

**Figure 1.**
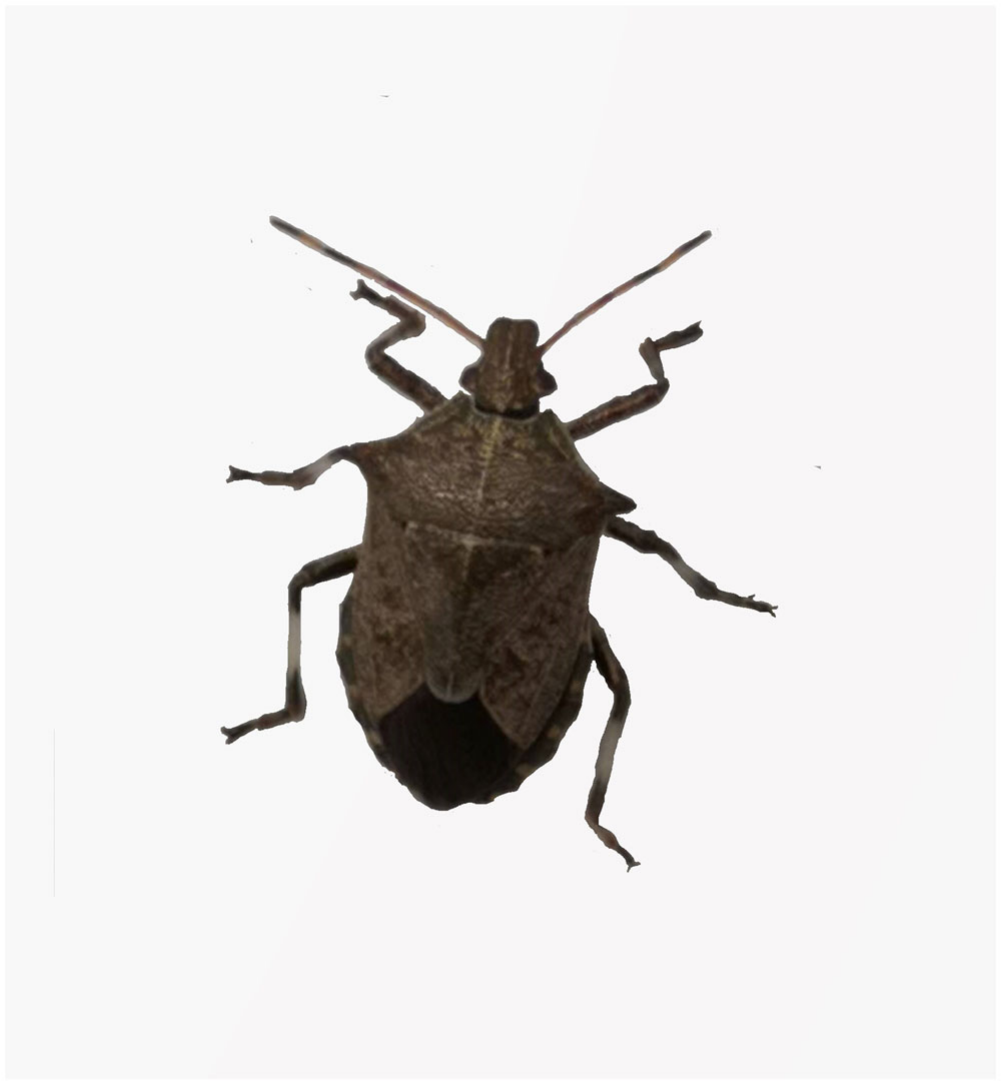
Adult of *P. lewisi.* Photos by Di-hui Yao.

The mitochondrial genome of *P. lewisi* was 18,123 bp long (GenBank accession OM140689) and consisted of 13 PCGs, 22 tRNA genes, two rRNA genes (*lrRNA* and *srRNA*), and one control region. The length of *lrRNA* and *srRNA* is 1,276 bp and 800 bp in length. The lengths of the 22 tRNA genes ranged from 63 bp (*trnC*) to 74 bp (*trnK*) (Figure 2).

**Figure 2.**
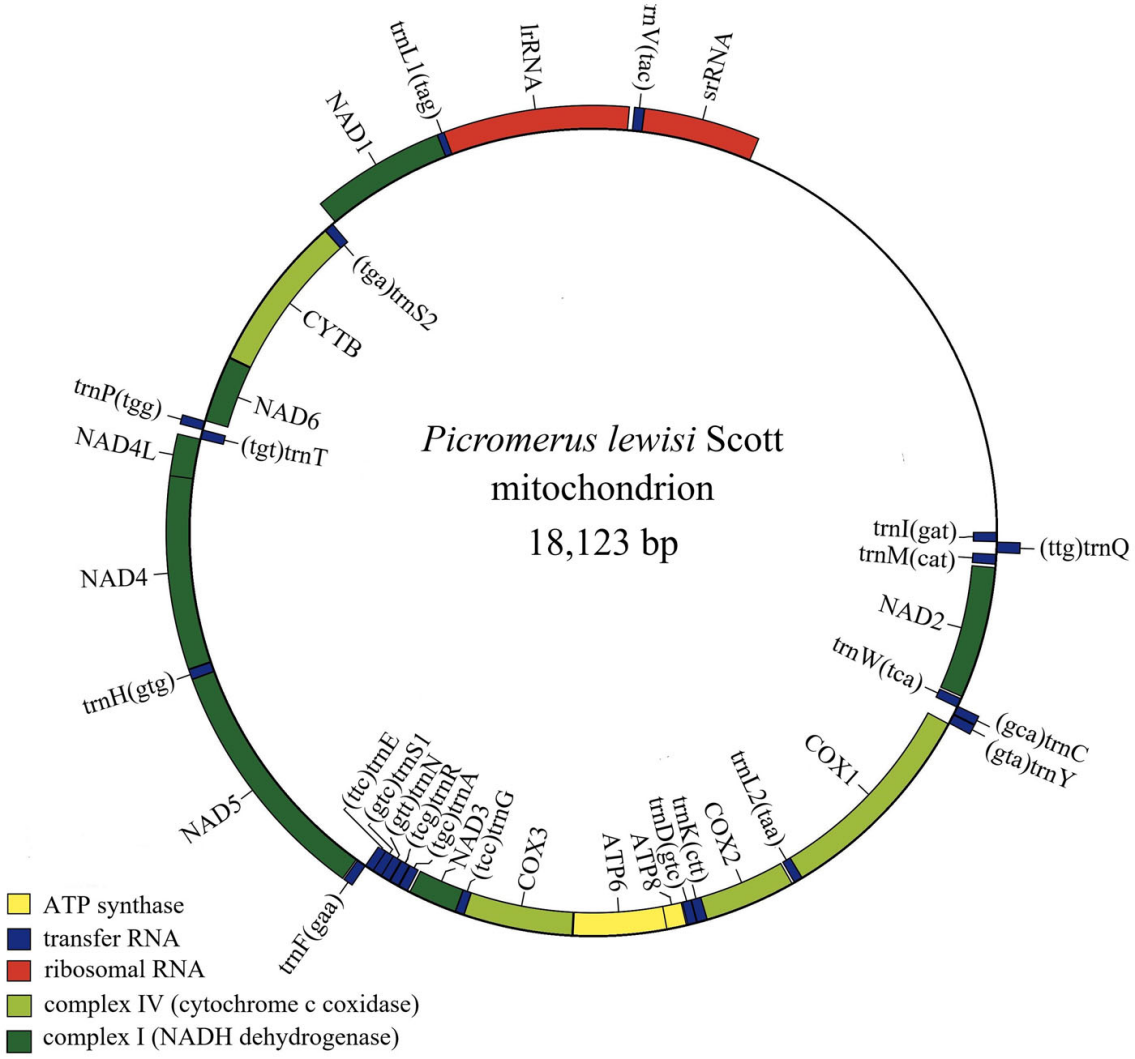
Genome map of *P. lewisi.* Mitochondrial genome map was drawn using the Chloroplot (https://irscope.shinyapps.io/Chloroplot/).

The overall base composition of the mitogenome was estimated as follows: A 40.9%, T 33.1%, C 15.3%, and 10.7 8.0%, with a high A + T content of 74.0%. Among the 13 PCGs, twelve PCGs started with a typical ATN codon: five with ATT (*NAD2, NAD4L, NAD5, NAD6, COX2*), four with ATG(*ATP6, COX3, NAD4, CYTB*), three with ATA(*ATP8, NAD1, NAD3*), whereas *COX1* started with TTG. Most of the PCGs terminated with the stop codon TAA, *CYTB* ended with stop codon TAG, whilst *COX2* uses a single T residue as an incomplete stop codon. The A + T content of 22 tRNA of *Picromerus lewisi* Scott ranged from 60.6% (*trnM*) to 89.1% (*trnL2*). The A + T content of *IrRNA* and *srRNA* are 76.6% and 76.1%, respectively.

Based on the concatenated amino acid sequences of 13 PCGs from 17 Panheteroptera species, PhyloSuite v1.2.2 (Zhang et al. 2020) was used for phylogenetic analysis, a phylogenetic tree was constructed using Bayesian inference (BI) in MrBayes (Huelsenbeck and Ronquist 2001). The iTOL webtool (https://itol.embl.de) (Letunic and Bork 2007) was used to view the phylogenetic tree. *Corythucha marmorata* and *Lygus pratensis* were used as outgroups. Our analysis positioned *P. lewisi* in a well-supported clade with *Eocanthecona thomsoni*. It was shown that *P. lewisi* has a closer relationship with *E. thomsoni* within Pentatomidae family, which accord with the traditional taxonomy. In conclusion, the complete mitogenome of *P. lewisi* is decoded in this study and provides essential and important DNA molecular data for further phylogenetic and evolutionary analysis for Pentatomidae (Figure 3).

**Figure 3.**
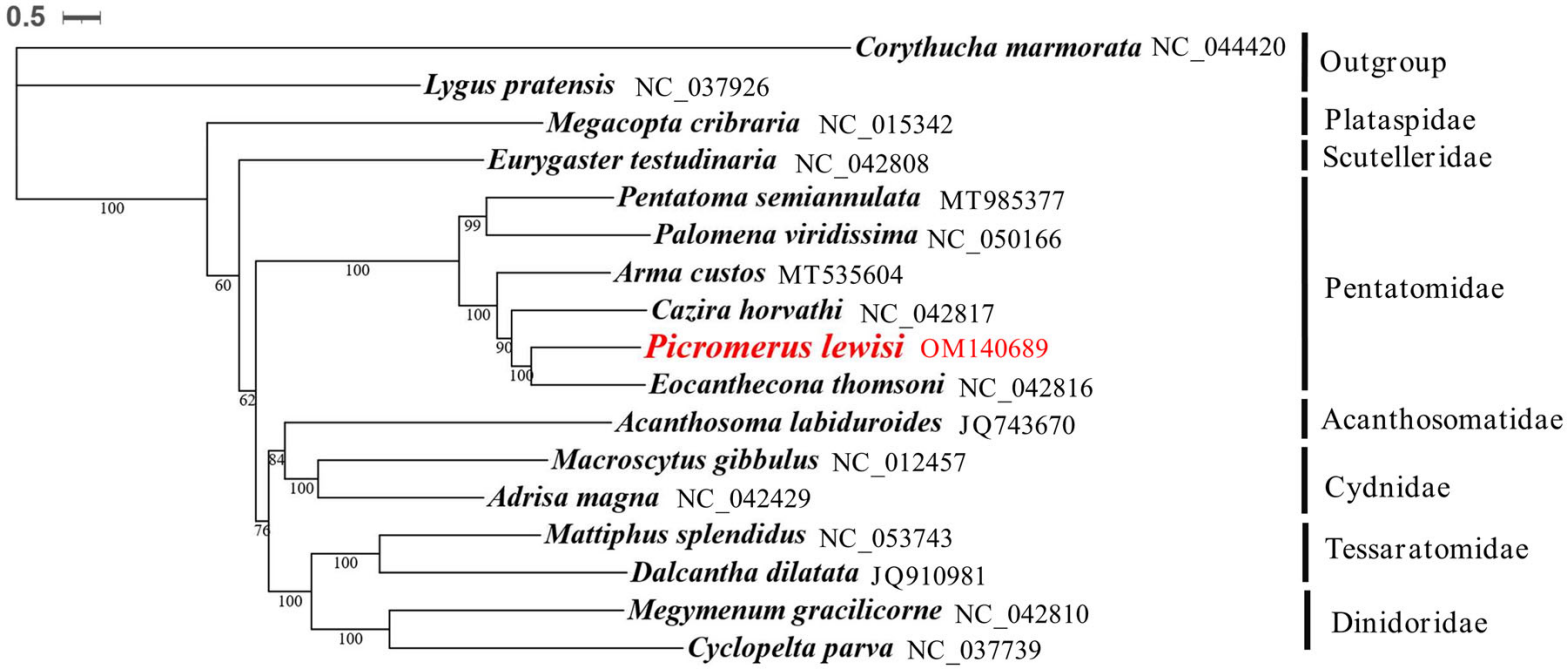
Phylogenetic relationships based on the 17 mitochondrial protein-coding gene sequences. Numbers on branches are Bootstrap support values (BS).

## Data Availability

The genome sequence data that support the findings of this study are openly available in GenBank of NCBI at (https://www.ncbi.nlm.nih.gov/) under accession no. OM140689. The associated BioProject, SRA, and BioSample numbers are PRJNA890215, SRR21891108, and SAMN31269902, respectively.
